# The Dangers of Potent Drugs in the Hands of Careless Invalids

**Published:** 1895-05

**Authors:** E. B. Jackson

**Affiliations:** Houston


For Texas Medical Journal.

### THE DANGERS OF POTENT DRUGS IN THE HANDS OF CARELESS INVALIDS.

E. B. JACKSON, M. D., HOUSTON.

SOME FEW weeks ago I was applied to by a lady who wished to have a needle removed from her forearm. She was addicted to the hypodermic use of morphine, through which practice the accident happened to her.

After removing the needle, I listened to her history, which dated years back. The history started with an account of uterine pain, closely followed by gastralgia and cardiac derangement, and finally by neuralgic headache which led up to her terrible habit. She was now suffering from dilatation, palpitation,—perhaps softening of the heart muscle,—and alarming anæmia.

Morphine no longer entirely relieved her attacks of pain, colic and syncope; so, seeing the salutary stimulating effect of the cocaine used in the needle extraction, she procured an ounce of a four per cent. solution. For her spells of cardiac pain, choking and unconsciousness, I had prescribed nitrite amyl, and directed her husband to use one to five drops on a kerchief given her by inhalation on the occasion of her attack. In his absence, she undertook to use a dose of cocaine, filling her syringe from the similar sized bottle of *amyl*, but used only five drops of the liquid. The pulse quickly jumped to 160, the face flushed crimson, the breathing became hurried, the sight dimmed, vertigo set up, and her heart almost ran away with her. Retaining consciousness, she prevailed upon her husband to administer a syringe full of the cocaine solution (30 m. 1¼ gr.), thinking to be made easy without fail, wherefore the deception in dose, as in reality she had hitherto used only ten minims to the dose; but the result was rapid and happy, the symptoms dispersing almost as quickly as they had appeared.

The next example which I report is, indeed, a comedy of errors. About one year ago I was treating a German lady, in her seventies, for "shingles." The band of eruptions was some three inches wide and extended half around the body. After cleansing the surface with a warm antiseptic wash, and touching all the parts which presented a weeping aspect with a weak solution of nitrate silver, and painting over all with collodion, I prescribed a castor oil emulsion containing thirty drops of laudanum to the tablespoonful every five or six hours, as required; also a wide roller of cotton wool to be covered thoroughly with corn starch; also collodion for reapplication upon the occasion of the next dressing. A grandchild two years old, was now brought in with an abscess three or four days old, under its chin, for which a poultice, simply, was prescribed. Before leaving, a grandson-in-law was prescribed for, for salivation, he having salivated himself; also a prescription of yellow oxide of mercury ointment was given to a daughter, for what seemed to be a phlyctenular conjunctivitis (such cases I never treat or even prescribe for, excepting those who are actually too poor to consult a specialist). The next day I found the daughter had rubbed her lids with the collodion, and rubbed her mother's side with the ointment, and had applied the cotton wool and corn starch to the baby's chin. Only the grandson-in-law carrying out directions to the letter. Five days later, when the grandmother was in great pain (and the shingles are painful without mercury ointment), she got the bottle of castor oil emulsion and took a dose every hour, until three were taken (90 drops laudanum), which gave her a good night's rest.

